# Low-Cost Ni-W Catalysts Supported on Glucose/Carbon Nanotube Hybrid Carbons for Sustainable Ethylene Glycol Synthesis

**DOI:** 10.3390/molecules29163962

**Published:** 2024-08-22

**Authors:** Rafael G. Morais, Lucília S. Ribeiro, José J. M. Órfão, Manuel Fernando R. Pereira

**Affiliations:** 1 LSRE-LCM—Laboratory of Separation and Reaction Engineering-Laboratory of Catalysis and Materials, Faculty of Engineering, University of Porto, Rua Dr. Roberto Frias, 4200-465 Porto, Portugal; rgm@fe.up.pt (R.G.M.); jjmo@fe.up.pt (J.J.M.Ó.); fpereira@fe.up.pt (M.F.R.P.); 2 ALiCE—Associate Laboratory in Chemical Engineering, Faculty of Engineering, University of Porto, Rua Dr. Roberto Frias, 4200-465 Porto, Portugal

**Keywords:** cellulose conversion, one-pot process, heterogeneous catalysis, hybrid glucose-based catalysts, ethylene glycol

## Abstract

The production of ethylene glycol (EG) from cellulose has garnered significant attention in recent years as an attractive alternative to fossil fuels due to the potential of cellulose as a renewable and sustainable feedstock. In this work, to the best of our knowledge, a series of low-cost Ni-W bimetallic catalysts supported on glucose/carbon nanotube hybrid carbons were synthesised for the first time and employed to transform cellulose into EG. Two different strategies were combined for the preparation of the carbons: the activation and addition of carbon nanotubes (CNTs) to obtain a hybrid material (AG-CNT). The catalytic conversion process proceeded through cellulose hydrolysis to glucose, followed by glucose retro-aldol condensation to glycolaldehyde and its subsequent hydrogenation to EG. Through the optimisation of the catalyst’s properties, particularly the metals’ content, a good synergistic effect of C-C bond cleavage and hydrogenation capabilities was assured, resulting in the highly selective production of EG. The balance between Ni and W active sites was confirmed to be a crucial parameter. Thus, total cellulose conversion (100%) was achieved with EG yields of 60–62%, which are amongst the best yields ever reported for the catalytic conversion of cellulose into EG via carbon-supported catalysts.

## 1. Introduction

Ethylene glycol (EG) is a valuable chemical due to its widespread use as a precursor for polyester fibres, plasticisers, cosmetics, antifreezes, and coolants. It is also used as a solvent or surfactant, amongst other applications [1,2]. Currently, EG is mainly produced from petroleum and coal. However, owing to its potential applications and environmental concerns, increasing attention has been paid to EG production from renewable biomass-based processes [3,4,5]. Lignocellulosic biomass is mainly composed of cellulose, hemicelluloses, and lignin, cellulose being the fraction that occupies the largest structure portion (40 to 80 wt.%) [3]. In this sense, cellulose’s conversion into high-value chemicals (e.g., EG) is the most attractive and viable method. The catalytic conversion of cellulose into EG comprises three steps, namely, hydrolysis, retro-aldol condensation (RAC), and hydrogenation [3]; therefore, the synthesis of multifunctional metal-supported catalysts is of paramount importance for this process. Additionally, the catalyst formulation should also consider using non-noble metals to decrease the catalyst cost and, thus, develop cost-competitive catalytic processes compared with the existing petroleum-based systems. Furthermore, carbon-based supports have received growing attention due to their large surface area and ease of surface modification. Also, hydrothermal carbonisation has been emerging as a promising method for preparing carbons with superior advantages when compared with commercial materials, such as lower cost, mild preparation conditions, the possibility of tailoring to specific functional groups, and textural properties, like a large surface area after carbonisation or activation [6,7]. Moreover, these carbon materials have good thermal, chemical, and mechanical stability, besides having an environmentally friendly nature [7].

Fukuoka and Dhepe’s pioneering work displayed the one-pot environmentally friendly conversion of cellulose into sugar alcohols, mainly sorbitol and mannitol, via supported metal catalysts [8]. Since then, many research groups have focused on this subject [9,10,11,12,13,14,15,16,17,18,19,20,21]. However, Ji et al. found out that the presence of tungsten species shifts the reaction pathway for EG production instead of C_6_ compounds, showcasing the capability of a nickel-promoted W_2_C catalyst in converting cellulose into EG by attaining a yield of 61% in just 30 min at 245 °C and 60 bar of H_2_ [22,23]. Then, under the same reaction conditions, Zheng et al. produced EG with a yield of around 62% via an activated carbon-supported Ru-W catalyst [24], while Zhao et al. obtained a 46% yield of EG via Ni-WP synthesised on activated carbon [25]. Afterwards, some groups managed to convert cellulose into EG using less severe reaction conditions. Ribeiro et al. reached an EG yield of around 42% via Ru-W catalysts supported on carbon nanotubes and glucose-derived carbons after 5 h at 205 °C and 50 bar of H_2_ [26,27], and, more recently, EG yields of around 50% via Ni-W catalysts supported on carbon nanotubes under the same conditions [28]. Moreover, a yield of EG close to 69% was reported by Huang et al. via a biochar-supported Ru-WO_x_ catalyst after 10 h at 220 °C and 50 bar of H_2_ [29]. More recently, also working at 220 °C and 50 bar of H_2_, yields of EG of 63 and 69% were attained after 2 h by Wu et al. [30] and Gao et al. [2], respectively, via Ni-W catalysts supported on glucose-derived carbons.

The existing literature emphasises the tremendous influence of a catalyst’s properties on its catalytic performance, and, as aforementioned, some works have proved that the presence of noble metals like Ru can be replaced with less-expensive metals (e.g., Ni) without decreasing the productivity of EG. Accordingly, the importance of using non-noble metal catalysts in further studies should be highlighted, as well as the development of low-cost carbon materials to be used as catalytic supports in the replacement of expensive materials like carbon nanotubes. Hence, in this work, low-cost Ni-W catalysts supported on glucose-derived carbon/carbon nanotube hybrids were synthesised and characterised using various techniques to explore their structural differences. Afterwards, the catalysts were evaluated in the one-pot conversion of cellulose to establish a structure–performance relationship. The EG yields obtained are amongst the best results reported to date from the environmentally friendly conversion of cellulose via carbon-supported catalysts. Therefore, in this paper, we report a promising catalyst with remarkable catalytic performance for the direct conversion of cellulose into EG in water media.

## 2. Results and Discussion

### 2.1. Material Characterisation

Microcrystalline and ball-milled cellulose have been extensively characterised elsewhere [31].

**Figure 1:**
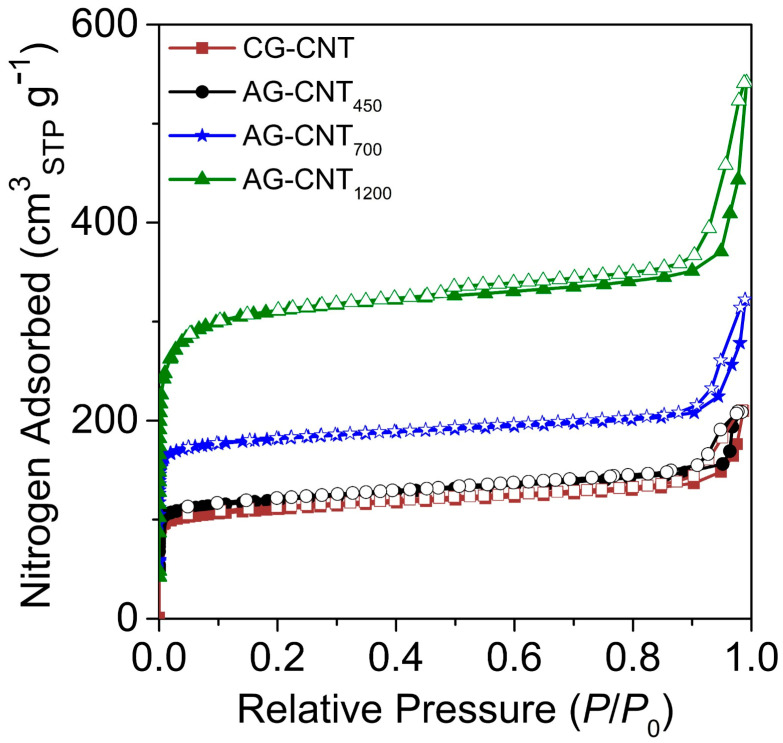
N_2_ adsorption–desorption isotherms of the hybrid supports.

The textural properties of the glucose-derived carbon/carbon nanotube hybrids were determined using the N_2_ adsorption–desorption isotherms exhibited in Figure 1, and the calculated parameters are displayed in Table 1.

All the prepared carbon supports were mainly microporous, as observed in their type I isotherms. In this series of samples, using an inert atmosphere (N_2_) during the thermal treatment led to a lower specific surface area (*S*_BET_) and micropore volume (*V*_µpores_) than the materials prepared under an atmosphere containing a physical activation agent (CO_2_). Regarding the latter samples, the temperature employed during the thermal treatment played an important role in the development of the supports’ porosity, as observed in samples AG-CNT_450_ and AG-CNT_700_, both treated for 2 h but at 700 °C and 900 °C, respectively. In this case, an increase in temperature resulted in a rise in the *S*_BET_ (465 vs. 727 m^2^ g^−1^) and *V*_µpores_ (0.150 vs. 0.250 cm^3^ g^−1^). Furthermore, by increasing the contact time between the carbon support and CO_2_ at 900 °C from 2 to 6 h (AG-CNT_1200_), a similar trend was observed in which the *S*_BET_ and *V*_µpores_ increased to 1220 m^2^ g^−1^ and 0.432 cm^3^ g^−1^, respectively.

The chemical bulk composition of the prepared carbons was analysed by the elemental analysis of the CNHS and O elements to further understand the modifications of each synthesis step. The EA results of the synthesised carbon supports are represented in Table 1. The carbon supports displayed differences in their carbon contents by varying each of the three thermal treatment parameters. The syntheses carried out under a CO_2_ atmosphere led to supports with higher C contents than those prepared under a N_2_ atmosphere, suggesting that the activating agent favours a relative carbon content rise. The variation in the thermal treatment temperature from 700 °C to 900 °C further increased the activated samples’ relative carbon contents. Regarding the contact time between CO_2_ and the carbon material, increasing the thermal treatment duration from 2 to 6 h further increased the carbon contribution to the total bulk composition.

The carbon supports were then used to incorporate Ni and W species. The N_2_ adsorption–desorption isotherms of these samples are exhibited in Figure S1, whereas the textural properties determined from those isotherms are represented in Table 2. The isotherms of the synthesised bimetallic catalysts supported on different hybrid carbons displayed a significant decrease in the specific surface area (*S*_BET_) and micropore volume (*V*_µpores_) compared with their respective carbon supports. Regarding the variation in the Ni and W contents, the *S*_BET_ and *V*_µpores_ decreased with the increase in the metals’ nominal amount, which was in agreement with the higher presence of metal species on the carbon support, which ultimately blocked the materials’ porosity.

The Ni content in the bimetallic samples, displayed in Table 2, was analysed by two techniques: ICP-OES and AAS. The results obtained by the different methods proved to be very similar, with a maximum 2.1 p.p. difference. The bimetallic samples obtained from different supports exhibited a trend in which the Ni content decreased with the use of CO_2_, an increase in the thermal treatment temperature, and a longer contact time between the activating agent and the carbon material. These results suggest that the samples became more chemically stable with the variations in the above-mentioned conditions for each of these three parameters. The samples prepared by varying the amount of Ni precursor impregnated on the carbon support also followed the expected trend and displayed a rise in the Ni content as the amount of nickel nitrate added increased. Overall, the Ni loading of all samples was slightly higher than the respective nominal values, suggesting a possible burn-off of the carbon support during the preparation steps. Due to equipment limitations, the W loading could not be assessed, but, taking into account the Ni content behaviour, the W content was assumed to be also slightly higher than the nominal values for all the catalysts.

**Figure 2:**
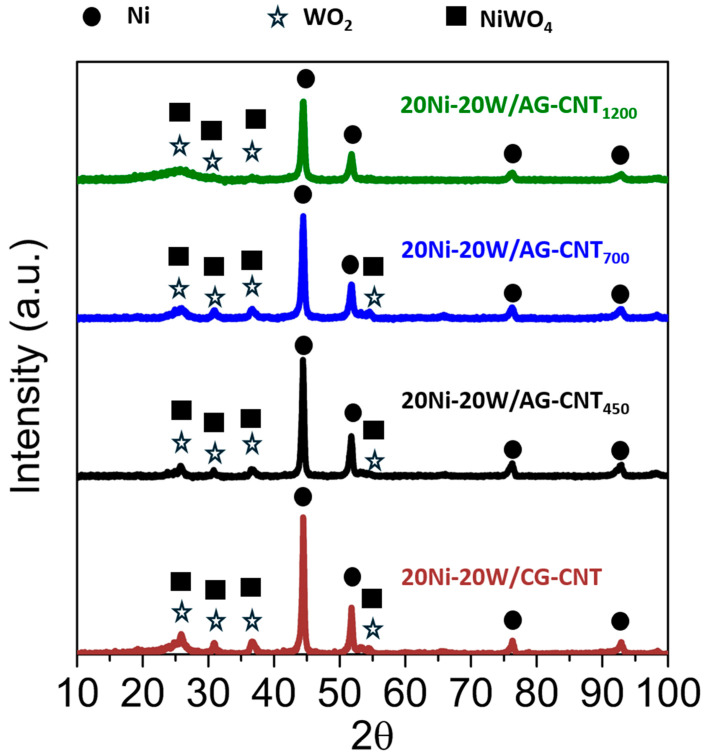
XRD patterns of the Ni-W bimetallic catalysts with different supports.

**Figure 3:**
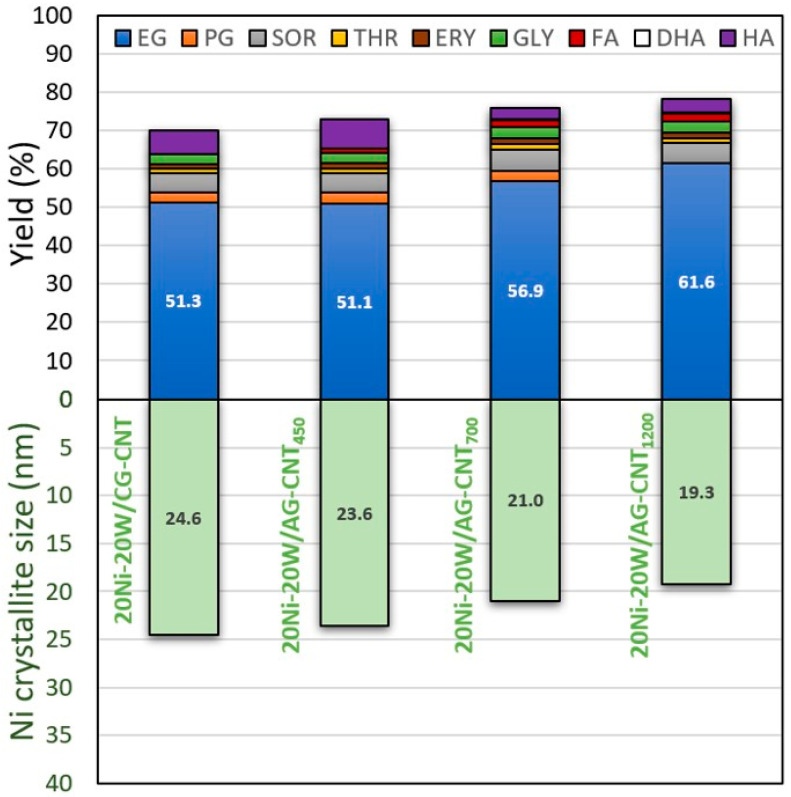
Effect of the catalyst properties on the distribution of products from cellulose conversion after 5 h. [EG: ethylene glycol; PG: propylene glycol; SOR: sorbitol; THR: threitol; ERY: erythritol; GLY: glycerol; FA: formic acid; DHA: dihydroxyacetone; HA: hydroxyacetone].

The bimetallic catalysts were analysed by XRD to study the Ni and/or W species present on the different supports after the thermal treatments under a H_2_ atmosphere. The obtained XRD spectra are displayed in Figure 2. The Ni peaks (ref. code 03-065-2865) at ca. 2θ = 44.5°, 51.8°, 76.4°, and 92.9° were observed in all samples analysed [32], which suggests that the use of a H_2_ atmosphere during the thermal treatments was efficient in the reduction in the Ni precursor. Considering the W precursor, peaks ascribed to WO_2_ (ref. code 00-032-1393) were detected in all catalysts at ca. 2θ = 25.8°, 31.6°, 36.8°, and 52.9° [33], which also partially overlapped with the NiWO_4_ peaks (ref. code 01-072-1189) exhibited at 24.0°, 30.9°, 36.6°, 52.4°, and 54.7° [34]. These results indicate that, regardless of the carbon support used, the reduction in the Ni and W precursors was similar in all materials. To further identify possible differences between the bimetallic catalysts prepared, the size of the Ni crystallites (Table 2) was calculated by the Scherrer equation [35]. The support obtained at 700 °C, using a CO_2_ atmosphere during the thermal treatment, favoured the formation of a slightly smaller crystallite size than when using an inert (N_2_) atmosphere (23.6 vs. 24.6 nm). Regarding the thermal treatment temperature, an increase from 700 °C to 900 °C during the synthesis of the support further decreased the crystallite size to 21.0 nm. Lastly, by prolonging the contact time of CO_2_ with the material from 2 to 6 h, a slight decrease in the crystallite size to 19.3 nm was observed. Moreover, amongst the four samples 20Ni-20W synthesised on different supports, the catalyst with a smaller Ni crystallite size (19.3 nm) was able to reach a higher yield of EG (61.6%), as discussed later (cf. Figure 3). Additionally, it is possible to infer that the Ni element was mainly present in all the catalysts in its metallic form (Ni^0^), with some traces of Ni^2+^ (NiWO_4_). Regarding W, it was mostly found in its +6 oxidation state (NiWO_4_) for all the samples prepared.

Furthermore, the analysed samples displayed Raman spectra with two distinct main peaks characteristic of carbon materials: the D band at ca. 1340 cm^−1^ associated with defects on the basal plane and the G band at ca. 1590 cm^−1^ ascribed to graphitic domains (Figure S2) [36].

### 2.2. Catalytic Properties for Cellulose Conversion

Apart from the operating conditions and substrate pre-treatment, two critical factors that can significantly impact the catalytic performance in the one-pot conversion of cellulose into ethylene glycol are the properties of the support and the metallic active phases’ content. Therefore, the type of support and metal content were assessed under the same conditions for cellulose conversion to obtain additional insight into the influence of these two parameters.

Firstly, the influence of the supports’ textural properties was evaluated (Figure 3 and Table S1). A total conversion of cellulose (100%) was achieved after 5 h, regardless of the catalyst used (Table S1, entries 3–6). Moreover, the catalyst supported on the carbonised hybrid (CG-CNT) allowed us to attain an outstanding EG yield of 51.3%, which was similar (51.1%) to that obtained via the catalyst supported on the activated hybrid with similar textural properties (AG-CNT_450_). However, regarding the three activated samples (AG-CNT_x_), it is possible to observe that the increase in the activating conditions (temperature and time) resulted in a decrease in the Ni crystallite size (from 23.6 to 21.0 and 19.3 nm), thereby augmenting the yield of EG (from 51.1 to 56.9 and 61.6%) (Figure 3). These results highlight the importance of the catalyst’s properties for the production of ethylene glycol directly from cellulose. Considering these results, AG-CNT_1200_ was the catalytic hybrid support selected for subsequent studies.

**Figure 4:**
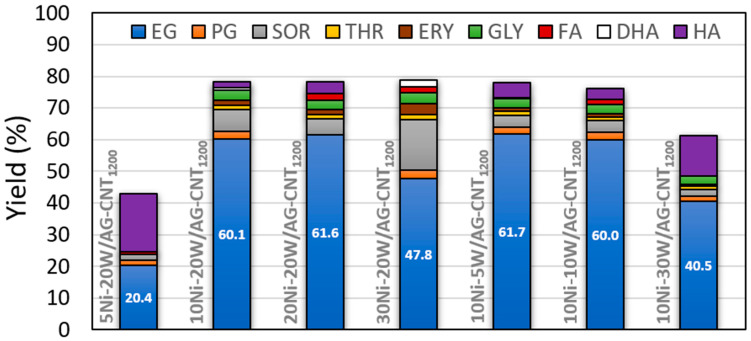
Distribution of products after 5 h of reaction by varying the nickel and tungsten contents. [EG: ethylene glycol; PG: propylene glycol; SOR: sorbitol; THR: threitol; ERY: erythritol; GLY: glycerol; FA: formic acid; DHA: dihydroxyacetone; HA: hydroxyacetone].

Afterwards, the effect of the metal loading was explored by changing the Ni and W nominal contents from 5 to 30 wt.% (Figure 4 and Table S1). The increase in the Ni loading from 5 to 10 and 20 wt.% resulted in a continuous increase in the yield of EG from 20.4 to 60.1 and 61.6%, respectively. However, despite the observed trend, further increasing the Ni loading from 20 to 30 wt.% was not beneficial for the production of EG, resulting in a yield drop from 61.6 to 47.8%. Moreover, unlike the behaviour observed when varying the Ni loading, the catalyst with the lowest W content was the one that allowed us to attain a higher EG yield (61.7%). At higher tungsten loadings, the yield was decreased by up to 40.5%, showing that the increase in the content of W negatively affected the yield of EG and instead favoured the production of HA (a 12.6% yield via 10Ni-30W/AG-CNT_1200_) (Table S1, entry 12).

**Figure 5:**
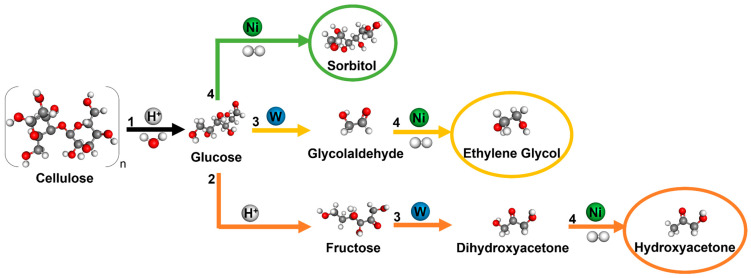
Simplified reaction pathway for the conversion of cellulose into EG, SOR, and HA [1—hydrolysis; 2—isomerisation; 3—retro-aldol condensation (RAC); 4—hydrogenation].

Yet, a deeper analysis of the results obtained can be conducted by looking at the possible reaction pathways of cellulose’s catalytic conversion. Figure 5 depicts the simplified routes that lead to the production of the main products herein obtained, including the respective intermediates. In this sense, the production of EG directly from cellulose is achieved after three subsequent steps: (i) the hydrolysis of cellulose to glucose, which can be catalysed by protons in situ generated reversibly from hot water; (ii) the retro-aldol condensation (RAC) of glucose to glycolaldehyde (GA), in which tungsten species play an important role cleaving glucose’s C-C bond to form GA; and, finally, (iii) the hydrogenation of GA to EG, promoted by hydrogenation active sites (e.g., Ru and Ni) (Figure 5: yellow route) [26,37]. However, parallel reactions can occur, such as the formation of sorbitol, which involves only two steps: the hydrolysis of cellulose to glucose, followed by its hydrogenation to sorbitol (Figure 5: green route) [15]. Also, another possible route could be the isomerisation of glucose to fructose, which further undergoes RAC to dihydroxyacetone and subsequent hydrogenation to hydroxyacetone (HA) (Figure 5: orange route).

Taking these possible reaction pathways into consideration, it can be observed that the production of EG was favoured in two situations: (i) Ni and W in a ratio of 1:1 (10Ni-10W/AG-CNT_1200_ and 20Ni-20W/AG-CNT_1200_); (ii) Ni and W in a ratio of 1:2 (10Ni-20W/AG-CNT_1200_) and 2:1 (10Ni-5W/AG-CNT_1200_) (Figure 4). Within these situations, the EG production was maximised to 60.0–61.7%, and the production of secondary products was minimised (e.g., 3.8–6.9% sorbitol and 1.9–4.7% hydroxyacetone) (Figure 4 and Table S1); thus, Figure 5’s yellow pathway was mainly followed. However, when the Ni content was significantly lower than that of W (i.e., Ni and W ratios of 1:4 for 5Ni-20W/AG-CNT_1200_ and 1:3 for 10Ni-30W/AG-CNT_1200_), the hydrogenation active sites were not sufficient to carry out the hydrogenation; instead, isomerisation took place, leading to the production of HA (12.6–18.4% yield) and other products (Table S1, entries 7 and 12). Moreover, the excess of W active sites seemed to divide into the two possible RACs (Figure 5: yellow and orange routes), since the production of EG appeared to occur in parallel with HA. Meanwhile, when the Ni content was significantly higher than the loading of W (i.e., a 10 p.p. difference between Ni and W for 30Ni-20W/AG-CNT_1200_), the excess of Ni provided dense hydrogenation actives sites that contributed directly to sorbitol production (Figure 5: green route), whose yield increased to 16.0%, thereby resulting in a decreased yield of EG (47.8%) (Figure 4 and Table S1). Furthermore, as expected, raising the Ni hydrogenation sites had a negative effect on the production of HA. Thus, the amount of Ni and W active sites must be well balanced to promote the production of EG. The catalysts that allowed us to maximise the EG yield up to 60.0–61.7% were 10Ni-10W/AG-CNT_1200_, 20Ni-20W/AG-CNT_1200_, 10Ni-5W/AG-CNT_1200_, and 10Ni-20W/AG-CNT_1200_. In fact, taking into consideration the standard deviation of the yields reported (±1.7%), it can be concluded that the four catalysts had similar performances. Thus, looking towards the need to develop a cost-competitive catalyst in comparison with the existing petroleum-based ones, the catalyst 10Ni-5W/AG-CNT_1200_ (61.7% EG yield) should be selected since it contains a lower metal content.

Additionally, Figure S3 shows the evolution of the obtained products with the reaction time in the presence of the 10Ni-5W/AG-CNT_1200_ catalyst. It is possible to observe an initial production of GA, which started decreasing after 1 h of reaction due to its further fast conversion to EG. Meanwhile, the yield of HA also started to increase slightly at the beginning of the reaction, but then it stabilised since the production of EG from GA was favoured. Also, the yield of EG rapidly increased within the first 3 h of the reaction (up to 57.8%) and presented a slight increase in the last 2 h (to 58.1 and 61.7% after 4 and 5 h, respectively). However, it is also possible to observe that there is still 22.1% of unidentified products. Due to the complex process of cellulose conversion, this could be attributed to the possible formation of gaseous products, water-soluble non-identified oligomers, or humins. Therefore, two additional experiments were carried out to better understand the reaction process: (i) only using the hybrid support (AG-CNT_1200_) and (ii) via a monometallic Ni catalyst supported on AG-CNT_1200_. The results can be found in Table S1 (entries 1–2). Briefly, a 100% conversion of cellulose could be attained via just the support (without any metal), but the yields of sugar alcohols were very low. Meanwhile, when Ni was incorporated into the support, a total conversion was, again, achieved, but the main product formed was sorbitol (29.6%). In this case, the Ni monometallic catalyst clearly favoured the parallel reaction related to sorbitol production (Figure 5: green route). Despite this, adding W to the catalyst formulation immediately changed the course of the reaction, leading to EG production instead of sorbitol (Figure 5: yellow route). Once again, this leads us to conclude that an appropriate balance between the amount of Ni and W active sites is fundamental to promote the production of EG. Furthermore, the reusability of 10Ni-5W/AG-CNT_1200_ was also evaluated for four successive runs, and the catalyst was remarkably stable, showing just a slight decrease in the yield of EG from 61.7 to 56.0% after the four cycles (Figure S4).

Regarding the support used, we previously tested Ni-W bimetallic catalysts supported on CNT [28] and glucose-derived carbons [38]. Here, we managed to surpass the results previously obtained using Ni-W/CNT (50.3 vs. 61.7%) under the same reaction conditions (Table 3). Meanwhile, the results previously obtained using Ni-W/AG (62.1%) were similar to the current results using Ni-W/AG-CNT. Despite the similarity of the results obtained, the addition of CNTs to the catalyst formulation had two advantages: (i) a lower content of metals (20%Ni-10%W/AG vs. 10Ni-5W/AG-CNT) and (ii) a more stable catalyst (at least up to four successive runs) (Figure S4).

Despite the difficulty of comparing the results obtained herein with those reported in the literature due to the different conditions applied, it is possible to verify that previous studies led to a maximum EG yield of 69.2% via carbon-supported catalysts [2] (Table 3). In this sense, due to the milder reaction conditions and lower metal contents used in this work compared with the literature (e.g., 10Ni-5W/AG-CNT_1200_), the yields of EG obtained herein (60.0–61.7%) are amongst the best reported for the sustainable one-pot conversion of cellulose into EG via carbon-supported catalysts.

## 3. Experimental Section

### 3.1. Chemicals

The chemicals were used as acquired, without further purification.

Microcrystalline cellulose was bought from Alfa Aesar (Havervill, MA, USA). D-glucose anhydrous (99%) and commercial multiwalled carbon nanotubes (CNTs) from NC3100 series (average diameter = 9.5 nm; average length = 1.5 μm; carbon purity > 95%), purchased from HiMedia (Modautal, Germany) and Nanocyl (Sambreville, Belgium), respectively, were used for the preparation of the carbon supports. The introduction of active phases on the carbon supports was carried out using two metal precursors: nickel (II) nitrate hexahydrate (Ni (NO_3_)_2_.6H_2_O, ≥99%) acquired from Merck (Darmstadt, Germany) and ammonium tungsten oxide hydrate ((NH_4_)_6_H_2_W_12_O_40_.xH_2_O, 99.99%) purchased from Alfa Aesar. Ultrapure water with a conductivity of 18.2 µS cm^−1^ (obtained in a Milli-Q Millipore System, Burlington, MA, USA) was used for the preparation of all the solutions.

### 3.2. Preparation of Materials

Microcrystalline cellulose was ball-milled prior to the reaction for 4 h at 20 Hz in a Retsch Mixer Mill MM200 (Haan, Germany) [39].

Glucose/CNT hybrids were synthesised by the hydrothermal polymerisation of the sugar followed by a thermal treatment to expand the resulting materials’ porosity [27]. In this synthesis, a Teflon-lined autoclave (stainless steel) was used to enclose a solution containing ultrapure water (85 wt.%), CNTs (0.3 wt.%), and glucose (14.7 wt.%). The mixture was then sonicated to ensure the full dissolution of the glucose and a homogeneous CNT dispersion. Afterwards, hydrothermal polymerisation was carried out at 180 °C for 12 h, forming a brown organic polymer that was then thoroughly washed with ultrapure water and dried at 100 °C overnight. The porosity of the material was then developed by carbonisation under a N_2_ atmosphere or activation under CO_2_ gas. The carbonisation was carried out at 700 °C using a gas flow rate of 100 cm^3^ min^−1^ for 2 h (CG-CNT sample), whereas the activation was performed using a gas flow rate of 80 cm^3^ g^−1^ min^−1^ under three different sets of temperature/time conditions: (i) at 700 °C for 2 h (AG-CNT_450_ sample), (ii) at 900 °C for 2 h (AG-CNT_700_ sample), or (iii) at 900 °C for 6 h (AG-CNT_1200_ sample). These samples were labelled according to the value of the surface area calculated (values in m^2^ g^−1^) (cf. Table 1).

Ni-W bifunctional catalysts were prepared on the abovementioned carbon supports. In this case, the Ni and W precursors were added dropwise to the carbon supports by incipient wetness co-impregnation to prepare catalysts with a nominal metal loading of 20 wt.% each. The wetted materials were then dried overnight and underwent a thermal treatment at 500 °C for 3 h under a N_2_ atmosphere followed by 3 h of reduction under a H_2_ atmosphere at the same temperature. All thermal treatments were performed using a fixed heating ramp of 10 °C min^−1^. These samples were named according to the nominal amount of Ni and W incorporated (20 wt.% each) and the carbon support used (samples 20Ni-20W/X, where X represents the carbon support). Furthermore, six samples were prepared by using the AG-CNT_1200_ carbon support and varying the Ni and W loadings (5, 10, and 30 wt.%), resulting in the samples 5Ni-20W/AG-CNT_1200_, 10Ni-20W/AG-CNT_1200_, 30Ni-20W/AG-CNT_1200_, 10Ni-5W/AG-CNT_1200_, 10Ni-10W/AG-CNT_1200_, and 10Ni-30W/AG-CNT_1200_, respectively.

### 3.3. Characterisation of the Supports and Catalysts

The elemental analyses (EAs) of these materials were performed in triplicate with a vario MICRO cube (CHNS) and a rapid OXY cube analyser (O) from Elementar (Langenselbold, Germany). The prepared catalysts underwent combustion at 1050 °C (for CHNS) and pyrolysis at 1450 °C (for O).

The quantification of the nickel loading of the bimetallic catalysts was achieved by (i) inductively coupled plasma–optical emission spectrometry (ICP-OES) using a Thermo Fisher Scientific iCAP 7000 spectrometer (Waltham, MA, USA) and (ii) atomic absorption spectroscopy (AAS) using a Unicam Solar 939 apparatus (Algés, Portugal). The catalysts were digested in a HNO_3_ solution for 15 min at 180 °C, which leached the Ni species prior to the analyses.

The textural properties of the catalysts were assessed by the N_2_ adsorption–desorption at −196 °C obtained using a Quantachrome Autosorb iQ_2_ automated gas sorption analyser (Boynton Beach, FL, USA). All samples underwent a 12 h vacuum degassing process at 150 °C prior to the analyses. The Brunauer–Emmet–Teller (BET) equation was used to calculate the specific surface area (*S*_BET_), whereas the *t*-method was used to calculate the external surface area (S_ext_) and the micropore volume (*V*_µpores_). The total pore volume (*V*_p_) was estimated from the amount of nitrogen adsorbed at the saturation point ($P/P_{0}$ = 0.99).

X-ray diffraction (XRD) was used to analyse the metallic catalysts’ crystalline phase composition with an X’Pert Pro diffractometer equipped with a PIXcel detector (Panalytical, Malvern, UK). These measurements were performed via a Bragg–Brentano arrangement at 45 kV and 40 mA, with Cu Kα radiation (λ = 1.541874 Å) at a scanning speed of 0.01° s^−1^.

The materials were also analysed by Raman spectroscopy at a 532 nm monochromatic wavelength with Alpha 300 equipment (WiTec, Ulm, Germany).

### 3.4. Catalysts’ Evaluation and Products Analysis

The catalytic performance was evaluated in a 1000 mL Parr Instruments (Moline, IL, USA) stainless-steel autoclave (USA Mod. 5120). Ball-milled cellulose (750 mg), the catalyst (300 mg), and deionised water (300 mL) were loaded into the autoclave, which was sealed and purged with nitrogen (99.999%) three times. Subsequently, the autoclave was heated up to the desired temperature (205 °C) at 2 °C min^−1^ under stirring (300 rpm). As soon as the target temperature was reached, the reaction was initiated by switching to 5 MPa of H_2_ (99.999%) and was conducted for 5 h with a periodical withdrawal of samples for analysis. At the end of the reaction, the catalyst was recovered from the liquid mixture by filtration, washed thoroughly with deionised water, and dried overnight at 100 °C. For the catalyst’s reusability evaluation, a small amount of the fresh catalyst (<5 wt.%) was added before each test to attain the desired catalyst weight (300 mg) due to mass loss during this process.

To determine the conversion of cellulose (X), total organic carbon (TOC) data were obtained with TOC-L Shimadzu equipment (Kyoto, Japan), and the conversion was determined using the following equation:

(1) $$X\left(\%\right)=\frac{\mathrm{moles}\;\mathrm{of}\;\mathrm{total}\;\mathrm{organic}\;\mathrm{carbon}\;\mathrm{in}\;\mathrm{the}\;\mathrm{resultant}\;\mathrm{liquid}}{\mathrm{moles}\;\mathrm{of}\;\mathrm{carbon}\;\mathrm{in}\;\mathrm{cellulose}\;\mathrm{charged}\;\mathrm{into}\;\mathrm{the}\;\mathrm{reactor}}\times 100\%\;=\frac{C_{TOC}}{m_{cellulose}\times \frac{6\times 12.01}{162.13}\times \frac{1000}{V}}\times 100\%$$

where $C_{TOC}$ is the concentration of carbon in the resultant liquid from the reaction (ppm), $m_{cellulose}$ is the mass of cellulose initially charged to the reactor (g), V is the reaction volume (L), 12.01 refers to the molar mass of carbon (g mol^−1^), and 162.13 refers to the molar mass of an anhydroglucose unit (C_6_H_10_O_5_) that composes cellulose (g mol^−1^).

The liquid products were identified and quantified by high-performance liquid chromatography (HPLC) using an Elite LaChrom HITACHI apparatus (Tokyo, Japan). The products’ separation was carried out by an Alltech (Lexington, KY, USA) OA-1000 ion exclusion column (300 × 6.5 mm; eluent: 0.005 M H_2_SO_4_ at 0.5 mL min^−1^) using a refractive index detector. Then, the products’ yields (*Y*) were calculated as the ratio between the number of moles of carbon in the product (measured by HPLC) and the number of moles of carbon in the cellulose initially loaded into the reactor.

## 4. Main Conclusions

A series of multifunctional Ni-W catalysts supported on hybrid carbons was tested for the one-pot conversion of cellulose into EG. The hydrothermal methodology provides a simple and effective method to synthesise catalysts with tailored properties. After an appropriate adjustment of the catalytic properties, the synergistic effect of C-C bond cleavage and hydrogenation was maximised, allowing the complete conversion of cellulose with remarkable EG yields of up to 62%. This result overcame previously reported studies that used carbon nanotube-supported Ru-W catalysts under the same conditions and is amongst the best results reported to date for the catalytic conversion of cellulose into EG via carbon-supported catalysts. Therefore, this work shows an efficient method for producing EG from cellulose using low-cost biomass-based carbons as part of the catalytic support.

## Figures and Tables

**Table 1:** Characterisation results of the glucose-derived carbon/carbon nanotube hybrid supports.

Sample	*S*_BET_ (m^2^ g^−1^)	*S*_ext_ (m^2^ g^−1^)	*V*_µpores_ (cm^3^ g^−1^)	*V*_p_ (cm^3^ g^−1^)	C (wt.%)	H (wt.%)	N (wt.%)	S (wt.%)	O (wt.%)
CG-CNT	431	68	0.142	0.32	93.3	1.4	0.0	0.0	5.3
AG-CNT_450_	465	82	0.150	0.32	94.4	1.2	0.0	0.0	4.4
AG-CNT_700_	727	70	0.250	0.50	94.7	0.6	0.0	0.0	4.7
AG-CNT_1200_	1220	111	0.432	0.83	95.7	0.4	0.0	0.0	3.9

**Table 2:** Characterisation results of the bimetallic catalysts.

Catalyst	*S*_BET_ (m^2^ g^−1^)	*S*_ext_ (m^2^ g^−1^)	*V*_µpores_ (cm^3^ g^−1^)	*V*_p_ (cm^3^ g^−1^)	Ni (wt.%) ^[a]^	Ni (wt.%) ^[b]^	Ni Crystallite Size (nm)
20Ni-20W/CG-CNT	281	66	0.085	0.26	32.6	32.6	24.6
20Ni-20W/AG-CNT_450_	191	66	0.052	0.21	29.3	29.3	23.6
20Ni-20W/AG-CNT_700_	219	88	0.055	0.34	23.9	24.1	21.0
20Ni-20W/AG-CNT_1200_	549	120	0.176	0.46	23.6	22.9	19.3
5Ni-20W/AG-CNT_1200_	821	109	0.277	0.57	4.6	5.9	n.d.
10Ni-20W/AG-CNT_1200_	692	101	0.236	0.57	10.0	11.2	n.d.
30Ni-20W/AG-CNT_1200_	245	125	0.051	0.30	37.5	35.4	n.d.
10Ni-5W/AG-CNT_1200_	n.d.	n.d.	n.d.	n.d.	15.8	n.d.	n.d.
10Ni-10W/AG-CNT_1200_	933	164	0.309	0.84	14.3	n.d.	n.d.
10Ni-30W/AG-CNT_1200_	794	98	0.274	0.49	12.1	n.d.	n.d.

^[a]^ Determined by ICP; ^[b]^ determined by AAS; n.d.—not determined.

**Table 3:** Comparison of the synthesised catalyst with other carbon-supported catalysts reported in the literature for the conversion of cellulose.

Catalyst	*T* (°C)	$P_{H_{2}}$ (bar)	*t* (h)	*X* (%)	*Y*_EG_ (%)	Reference
0.4Ru-30W/CNT	205	50	3	100	42.4	[26]
0.4Ru-30W/CG	205	50	5	100	42.1	[27]
5Ru-25W/AC	245	60 ^[a]^	0.5	100	61.7	[24]
5Ru-40WO_x_/BC	220	50	10	99.8	68.8	[29]
2Ni-30W_2_C/AC	245	60 ^[a]^	0.5	100	61.0	[23]
2Ni-20WP/AC	245	60 ^[a]^	0.5	100	46.0	[25]
20Ni-20W/CNT	205	50	5	100	50.3	[28]
9Ni-13.5W/GC_850_	220	50 ^[a]^	2	100	69.2	[2]
9Ni-12WO_x_/GC_700_	220	50 ^[a]^	2	100	63.0	[30]
20Ni-10W/AG_850_	205	50	5	100	62.1	[38]
10Ni-5W/AG-CNT_1200_	205	50	5	100	61.7	This work

^[a]^ Measured at room temperature.

## Data Availability

The original contributions presented in this study are included in this article/the Supplementary Material. Further inquiries can be directed to the corresponding author.

## Supplementary Materials

The following supporting information can be downloaded at https://www.mdpi.com/article/10.3390/molecules29163962/s1. Figure S1: N_2_ adsorption–desorption isotherms of the Ni-W bimetallic catalysts; Figure S2: Raman spectra of the Ni-W bimetallic catalysts; Table S1: Catalytic results of cellulose conversion and yield of products; Figure S3: (a) Time-dependent product formation via 10Ni-5W/AG-CNT_1200_ and (b) zoom of 0–10% yield range; Figure S4: Reusability tests of 10Ni-5W/AG-CNT_1200_.
