# Supplementary material for: Low-Cost Ni-W Catalysts Supported on Glucose/Carbon Nanotube Hybrid Carbons for Sustainable Ethylene Glycol Synthesis
Source: Molecules. 2024 Aug 22;29(16):3962. doi: 10.3390/molecules29163962 (PMC11357644; doi:10.3390/molecules29163962)
Supplement: Supplementary file 1 [file molecules-29-03962-s001.zip › molecules-3163434-supplementary.pdf]

# Supporting Information

## Low-cost Ni-W catalysts supported on glucose/carbon nanotube hybrid carbons for sustainable ethylene glycol synthesis

Rafael G. Morais[1,2,†], Lucília S. Ribeiro[1,2,†,*], José J. M. Órfão[1,2], Manuel Fernando R. Pereira[1,2]

rgm@fe.up.pt, lucilia@fe.up.pt, jjmo@fe.up.pt, fpereira@fe.up.pt

[1] LSRE-LCM – Laboratory of Separation and Reaction Engineering - Laboratory of Catalysis and Materials, Faculty of Engineering, University of Porto, Rua Dr. Roberto Frias, 4200-465 Porto, Portugal

[2] ALiCE – Associate Laboratory in Chemical Engineering, Faculty of Engineering, University of Porto, Rua Dr. Roberto Frias, 4200-465 Porto, Portugal

†Both authors contributed equally to this work

*Corresponding author: Lucília S. Ribeiro, lucilia@fe.up.pt, Phone: +351 220 414 922

### 1. Characterization of catalysts

The $N_2$ adsorption-desorption isotherms of the synthesised Ni-W catalysts supported on the hybrid carbons are shown in Figure S1.

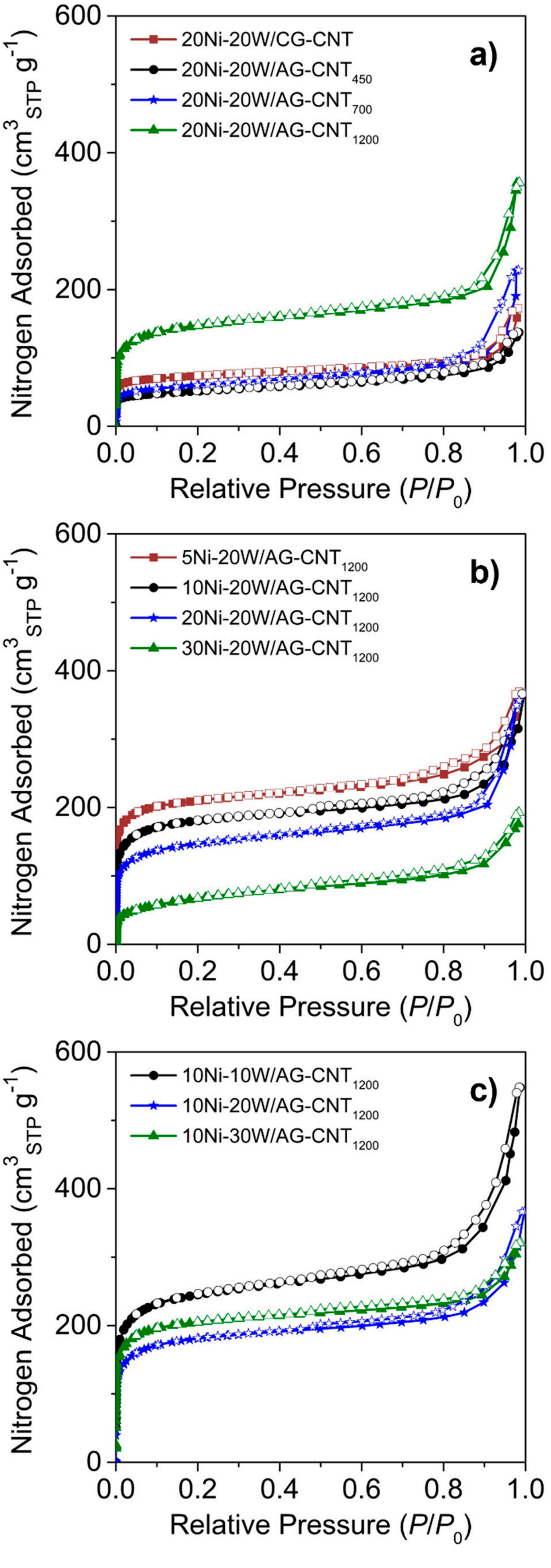


*Figure S1 – $N_2$ adsorption-desorption isotherms of the Ni-W bimetallic catalysts with different a) support, b) Ni content and c) W content.*

Figure S2 displays the Raman spectra of the synthesized catalysts.

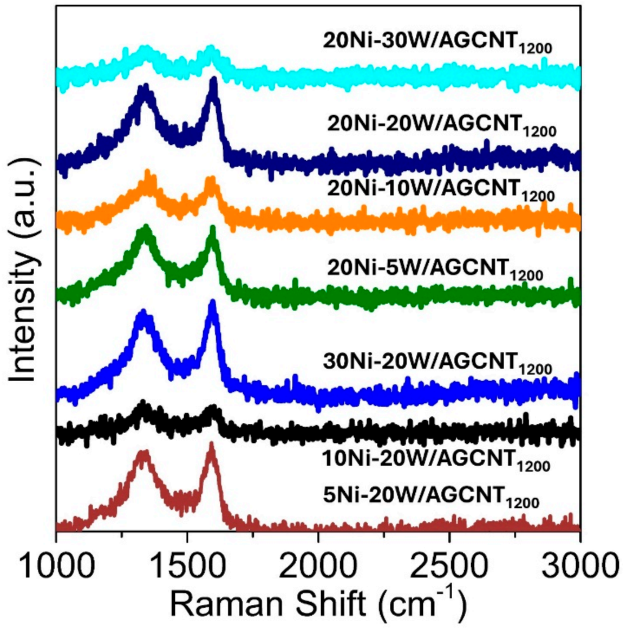


*Figure S2 – Raman spectra of the Ni-W bimetallic catalysts.*

## 2. Catalytic tests

The catalytic results obtained are presented in Table S1. Entries 1 and 2 display the results obtained in two additional tests: 1) using the hybrid support without the addition of the metal phase (AG-$CNT_{1200}$), and 2) using a monometallic Ni catalyst.

*Table S1 – Catalytic results of cellulose conversion and yield of products[a].*

| Entry | Catalyst | $X$ (%) | Yields ± 1.7 [b] (%) | | | | | | | | | |
|---|---|---|---|---|---|---|---|---|---|---|---|---|
| | | | EG | PG | SOR | THR | ERY | GLY | FA | DHA | HA | Others |
| 1 | AG-$CNT_{1200}$ | 100 | 8.2 | 3.8 | 1.9 | 0.0 | 0.0 | 8.5 | 1.7 | 0.0 | 0.0 | 75.9 |
| 2 | 20Ni/AG-$CNT_{1200}$ | 100 | 10.0 | 2.7 | 29.6 | 1.4 | 3.0 | 6.5 | 0.0 | 7.3 | 0.0 | 39.5 |
| 3 | 20Ni-20W/CG-CNT | 100 | 51.3 | 2.5 | 5.1 | 1.2 | 1.2 | 2.6 | 0.0 | 0.0 | 6.2 | 29.9 |
| 4 | 20Ni-20W/AG-$CNT_{450}$ | 100 | 51.1 | 2.8 | 5.0 | 1.2 | 1.3 | 2.8 | 1.1 | 0.0 | 7.6 | 27.1 |
| 5 | 20Ni-20W/AG-$CNT_{700}$ | 100 | 56.9 | 2.6 | 5.6 | 1.3 | 1.6 | 2.8 | 1.9 | 0.2 | 2.9 | 24.2 |
| 6 | 20Ni-20W/AG-$CNT_{1200}$ | 100 | 61.6 | 0.0 | 5.1 | 1.3 | 1.4 | 3.0 | 2.1 | 0.1 | 3.6 | 21.8 |
| 7 | 5Ni-20W/AG-$CNT_{1200}$ | 100 | 20.4 | 1.5 | 1.8 | 0.0 | 0.0 | 0.0 | 0.8 | 0.0 | 18.4 | 57.1 |
| 8 | 10Ni-20W/AG-$CNT_{1200}$ | 100 | 60.1 | 2.5 | 6.9 | 1.3 | 1.6 | 3.1 | 0.0 | 0.9 | 1.9 | 21.7 |
| 9 | 30Ni-20W/AG-$CNT_{1200}$ | 100 | 47.8 | 2.6 | 16.0 | 1.5 | 3.5 | 3.3 | 2.1 | 1.9 | 0.0 | 21.3 |
| 10 | 10Ni-5W/AG-$CNT_{1200}$ | 100 | 61.7 | 2.1 | 3.9 | 1.4 | 0.9 | 2.9 | 0.0 | 0.3 | 4.7 | 22.1 |
| 11 | 10Ni-10W/AG-$CNT_{1200}$ | 100 | 60.0 | 2.2 | 3.8 | 1.2 | 0.9 | 2.9 | 1.6 | 0.0 | 3.5 | 23.9 |
| 12 | 10Ni-30W/AG-$CNT_{1200}$ | 100 | 40.5 | 1.5 | 2.3 | 1.1 | 0.5 | 2.7 | 0.0 | 0.0 | 12.6 | 38.8 |

[a] Reaction conditions: 750 mg ball-milled cellulose, 300 mg catalyst, 300 mL $H_2O$, 205 ºC, 50 bar $H_2$, 300 rpm, 5 h.

[b] EG: ethylene glycol; PG: propylene glycol; SOR: sorbitol; THR: threitol; ERY: erythritol; GLY: glycerol; FA: formic acid; DHA: dihydroxyacetone; HA: hydroxyacetone.

Figure S3 shows the evolution of cellulose conversion and products distribution with the reaction time, using 10Ni-5W/AG-$CNT_{1200}$.

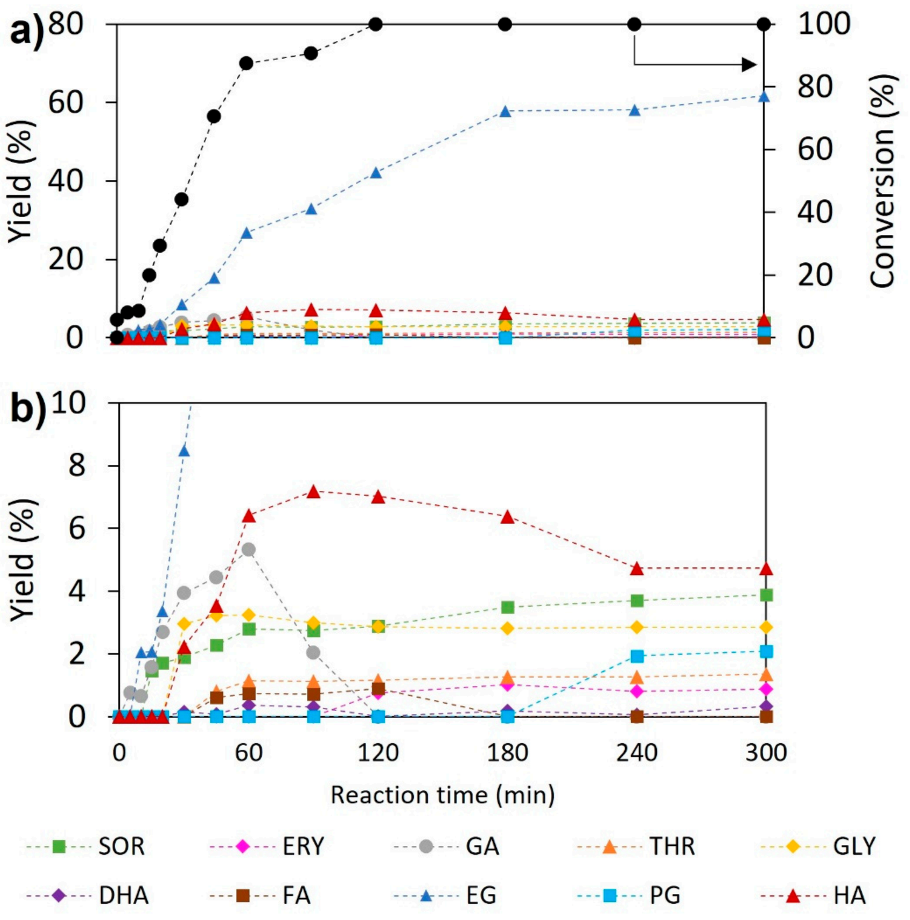


*Figure S3 – a) Time-dependent product formation over 10Ni-5W/AG-CNT$_{1200}$ and b) zoom of 0-10 % yield range. [EG: ethylene glycol; PG: propylene glycol; SOR: sorbitol; THR: threitol; ERY: erythritol; GA: glycolaldehyde; GLY: glycerol; FA: formic acid; DHA: dihydroxyacetone; HA: hydroxyacetone]*

Figure S4 shows the results obtained for reusability tests of 10Ni-5W/AG-$CNT_{1200}$.

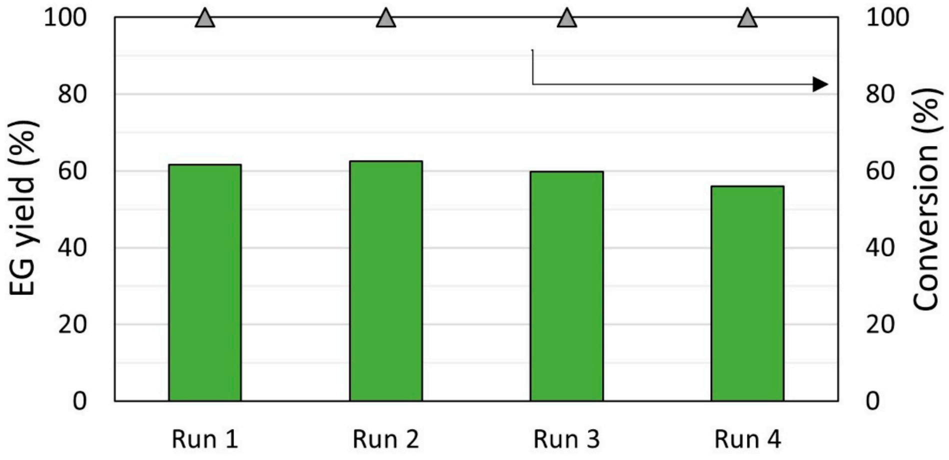


*Figure S4 – Reusability tests of 10Ni-5W/AG-$CNT_{1200}$. Reaction conditions: 750 mg ball-milled cellulose, 300 mg 10Ni-5W/AG-$CNT_{1200}$, 300 mL $H_2O$, 205 ºC, 50 bar $H_2$, 300 rpm, 5*
